# One-Step Solvothermal Synthesis of Ni Nanoparticle Catalysts Embedded in ZrO_2_ Porous Spheres to Suppress Carbon Deposition in Low-Temperature Dry Reforming of Methane

**DOI:** 10.1186/s11671-022-03683-7

**Published:** 2022-04-18

**Authors:** Meiliefiana Meiliefiana, Tsuzumi Nakayashiki, Emi Yamamoto, Kahoko Hayashi, Masataka Ohtani, Kazuya Kobiro

**Affiliations:** 1 Present Address: School of Environmental Science and Engineering, Kochi University of Technology, 185 Miyanokuchi, Tosayamada, Kochi 782-8502 Japan; 2 Laboratory for Structural Nanochemistry, 185 Miyanokuchi, Tosayamada, Kochi 782-8502 Japan

**Keywords:** Zirconia composite, Nickel catalyst, Low-temperature dry reforming of methane, One-pot solvothermal synthesis, Suppression of carbon deposition

## Abstract

Ni nanoparticle catalysts embedded in ZrO_2_ porous spheres and ZrO_2_ porous composite spheres, SiO_2_-ZrO_2_, MgO-ZrO_2_, and Y_2_O_3_-ZrO_2_, with 83−115 nm diameter and 167–269 m^2^/g specific surface area were prepared by a one-pot and one-step solvothermal reaction from precursor solutions consisting of Ni(NO_3_)_2_‧6H_2_O, Zr(O^*n*^Bu)_4_, and acetylacetone in moist ethanol combined with either Si(OEt)_4_, magnesium acetylacetate, or Y(O^*i*^Pr)_3_. The obtained Ni catalysts have high specific surface areas of 130–196 m^2^/g, even after high-temperature reduction by H_2_ at 450 °C for 2 h. They were utilized as catalysts for low-temperature dry reforming of methane (DRM) at 550 °C to suppress carbon deposition on Ni nanoparticles. The Ni catalysts embedded in SiO_2_-ZrO_2_ and Y_2_O_3_-ZrO_2_ demonstrated high catalytic activity and long stability in the reaction. Moreover, carbon deposition on Ni nanoparticles in the DRM reaction was effectively suppressed in when using the SiO_2_-ZrO_2_ and Y_2_O_3_-ZrO_2_ composites.

**Graphical abstract:**

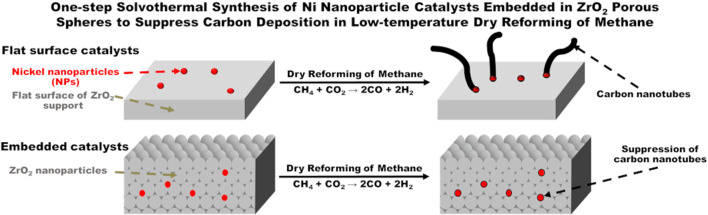

**Supplementary Information:**

The online version contains supplementary material available at 10.1186/s11671-022-03683-7.

## Introduction

Metal nanoparticles (NPs) have excellent catalytic activity in heterogeneous catalysis [1]. Numerous methodologies have been developed to enhance their stabilities and achieve long lifetimes for catalytic active sites by optimizing the combination of metal NP size and catalyst support [2]. However, the metal NPs on the surface of the catalyst supports still tend to aggregate and sinter at high temperatures and after prolonged practical use. Much effort has been devoted to preventing the aggregation and sintering of metal NPs on the support surface [3]. Embedding or encapsulation of NPs into porous metal oxides is an effective approach for improving catalyst stability by physical separation of the metal NPs [4]. Several approaches for embedding or encapsulating noble metal NPs in metal oxide supports have been reported. For example, Liu et al. reported a Pt NP catalyst embedded in wide-mouthed compartments tailored on a SiO_2_ support by a reduction method as a sintering resistance NP system [5]. Liu et al. prepared an encapsulation strategy for Au NPs in a permeable TiO_2_ thin layer by the deposition–precipitation method, resulting in excellent activity and stability for catalytic CO oxidation [6]. Xiao et al. prepared an embedded NP alloy from intermetallic PtFe alloys supported on carbon [7].

In this context, our group has developed a simple synthetic method for submicron-sized monodisperse porous metal oxides such as SiO_2_, TiO_2_, ZrO_2_, and CeO_2_ by a one-pot and single-step solvothermal approach. These materials are called micro-/meso-porously architected roundly integrated metal oxides (MARIMOs) [8]. For example, the TiO_2_ MARIMO consists of ca. 5 nm primary particles with large specific surface areas exceeding 300 m^2^/g, concomitant with an almost perfect spherical morphology [9]. The MARIMOs were used as a support for the Au NP catalysts. The Au NP catalyst prepared by the deposition–precipitation method (Au/TiO_2_) demonstrated excellent heat tolerance and durability for highly exothermic CO oxidation, while the nano-concave-convex surface of TiO_2_ MARIMO prevented well-dispersed Au NPs from migrating and sintering. As a result, the Au/TiO_2_ MARIMO catalyst exhibited low-temperature activity and long-term stability compared to that of Au NP catalysts supported on commercially available TiO_2_. We have also succeeded in synthesizing perfectly monodisperse ZrO_2_ porous spheres with large specific surface areas (244 m^2^/g) using a similar one-pot and single-step solvothermal approach [10]. The ZrO_2_ porous spheres were converted to a Ni/ZrO_2_ catalyst that demonstrated superior catalytic activity and heat tolerance for eminently exothermic CO_2_ methanation by the impregnation method. Similarly, composite metal oxide porous spheres were easily prepared by slightly modified one-pot and single-step solvothermal reactions [11–13]. When a Ru catalyst was applied on a SiO_2_-CeO_2_ support composite (Ru/SiO_2_-CeO_2_) prepared by impregnation, it exhibited low-temperature activity as well as long-term stability for CO_2_ methanation [14].

Meanwhile, the dry reforming of methane (DRM, Eq. 1) is a promising reaction for recycling CO_2_ [15]. Ni catalysts supported on (metal) oxides are commonly used in the DRM, although carbon deposition on Ni NPs is a serious disadvantage. To avoid the formation of carbon species, the reaction is usually performed at high temperatures, such as 700 °C. However, the sintering and aggregation of the Ni NPs as well as the (metal) oxide supports occurred concurrently, which was a serious drawback. Furthermore, according to the mechanism of carbon deposition in the DRM, it is possible for the exposed Ni NPs on the flat surface of the support to react with the excess methane molecules during the reaction, resulting in the accumulation of carbon atoms as nickel carbide, causing carbon deposition.

**Fig. 1:**
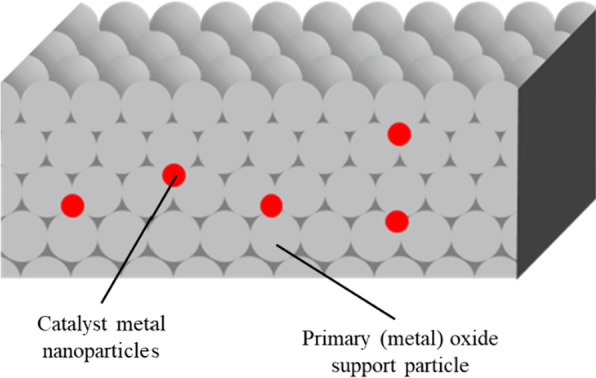
Schematic of embedded nanometal in gaps of primary (metal) oxide support nanoparticles

To avoid the carbon deposition, several approaches have been reported: Liu et al. prepared a core-shell catalyst with Ni-ZrO_2_@microporous SiO_2_ structure [16], Peng et al. fabricated a unique catalyst with Ni NPs confined on a dendric mesoporous SiO_2_ [17], Lin et al. also developed a core-shell catalyst with Ni-CeO_2_@microporous SiO_2_ structure [18], and Liu et al. reported an intermetallic alloy nano catalyst (In_x_Ni@SiO_2_) [19]. In this context, we hypothesized that metal NPs embedded into support (metal) oxides would physically suppress the formation of carbon species (Fig. 1). To demonstrate our hypothesis, we designed a new synthetic approach to embed Ni NPs into (composite) metal oxide supports by means of a new one-pot and single-step solvothermal technique.

1 $${\text{CH}}_{4} + {\text{CO}}_{2} \to 2{\text{CO}} + 2{\text{H}}_{2}$$

In this study, ZrO_2_ porous spheres were selected as catalyst supports because of their high heat tolerance. A SiO_2_-ZrO_2_ composite was selected as a catalyst support to extend the surface area, the MgO-ZrO_2_ composite was chosen as a catalyst support in anticipation of positive acid–base interactions between CO_2_ and MgO [20, 21], and Y_2_O_5_-ZrO_2_ functioned as a long-lifetime catalyst support by suppressing the sintering of the catalyst support.

## Methods

### Reagents

85% zirconium butoxide 1-butanol solution, nickel (II) nitrate hexahydrate, and tetraethoxysilane were purchased from Fujifilm Wako Chemical Corporation. Yttrium isopropoxides, acetylacetone, and magnesium acetylacetonate were purchased from Tokyo Chemical Industry Corporation. Ethanol was purchased from Kishida Chemical Corporation. UEP-100 ZrO_2_ NPs were obtained from Daiichi Kigenso Kagaku Kogyo Co., Ltd. All reagents were used without further purification.

### Catalyst Preparation

A precursor solution including 85 wt% zirconium butoxide in 1-butanol (3.20 g, 7.09 mmol), acetylacetone (50 mL, 0.48 mmol), and Ni(NO_3_)_2_·6H_2_O (500 mg, 1.68 mmol) in ethanol (35 mL) was heated in an SUS-316 stainless steel autoclave (MMS-5000, OM LAB-TECH Co., Ltd.) at 250 °C for 60 min, and the quantity of Ni salt was adjusted to Ni/support metal oxide(s) = 10/90 (*wt*/*wt*). The reactor was then cooled to room temperature. The obtained solid was centrifuged at 10,000 rpm for 15 min at 25 °C and washed three times with methanol. The solid was dried under vacuum for 20 h at room temperature to yield a powdery product. The product, denoted *L*-Ni@ZrO_2_, was calcined at 300 °C for 2 h in air. “*L*” and “@” represent “large” and “obtained by one-pot solvothermal method,” respectively. Ni catalysts supported on SiO_2_-ZrO_2_, MgO-ZrO_2_, and Y_2_O_3_-ZrO_2_ composite porous spheres were prepared as follows: tetraethoxysilane (156 μL, 0.703 mmol), magnesium acetylacetonate (155 μL, 0.699 mmol), or yttrium isopropoxide (18.6 mg, 0.700 mmol) was dissolved in ethanol (85 mL). 85 wt% zirconium butoxide solution in 1-butanol (2.84 g, 6.29 mmol), acetylacetone (50 mL, 0.48 mmol), and an ethanol (35 mL) solution of Ni(NO_3_)_2_·6H_2_O (445 mg, 1.53 mmol) were added to the solution successively, to produce a precursor solution, in which the Si, Mg, or Y content was adjusted to (Si, Mg, or Y)/Zr = 1/9 (mol/mol). Similar solvothermal treatment of the precursor solutions afforded Ni catalysts supported on the porous composite spheres, SiO_2_-ZrO_2_, MgO-ZrO_2_, and Y_2_O_3_-ZrO_2_, which are denoted *L*-Ni@SiO_2_-ZrO_2_, *L*-Ni@MgO-ZrO_2_, and *L*-Ni@Y_2_O_3_-ZrO_2_, respectively.

Ni catalysts supported on small porous spheres were obtained according to a procedure similar to that mentioned above, except for the addition of water (3 mL) to the precursor solutions. The prepared small catalysts are referred to as *S*-Ni@ZrO_2_, *S*-Ni@SiO_2_ − ZrO_2_, *S*-Ni@MgO − ZrO_2_, and *S*-Ni@Y_2_O_3_ − ZrO, in which “*S*” represents “small.” Reference Ni catalysts supported on UEP-100 ZrO_2_ and ZrO_2_ porous spheres were prepared by the impregnation method. A mixture of 131 mg (0.451 mmol) Ni(NO_3_)_2_·6H_2_O and 500 mg UEP-100 ZrO_2_ or ZrO_2_ porous spheres in 12.5 mL of water was mixed well using a planetary centrifugal mixer (THINKY AR-100) for 2 h. The obtained powder was dried in air at 80 °C for 2 h and then calcined in air at 300 °C for 2 h to produce a Ni catalyst, *U*-Ni/ZrO_2_ or *L*-Ni/ZrO_2_, where "*U*" and “/” represent "UEP-100" and “obtained by impregnation method,” respectively.

### Catalyst Characterization

Transmission electron microscopy (TEM) images were obtained using a JEOL JEM-2100F microscope. Energy-dispersive X-ray (EDX) spectroscopy was performed using an Oxford INCA X-Max 80 EDX spectrometer with the TEM instrument. Scanning electron microscopy (SEM) images were obtained using a Hitachi SU8020 FE-SEM. Inductively coupled plasma optical emission spectroscopy (ICP-OES) was performed using a Hitachi High-Tech Science PS3520UV-DD spectrometer. Elemental analysis was carried out on a Malvern Panalytical Epsilon 1 X-ray fluorescence (XRF) spectrometer. Nitrogen adsorption/desorption experiments were conducted using a BEL BELSORP mini II instrument. The specific surface area was calculated using the Brunauer–Emmett–Teller method using the obtained nitrogen adsorption–desorption isotherms. The crystalline phases of the catalysts were identified by X-ray diffraction (XRD) on a Rigaku SmartLab diffractometer using Cu-Kα radiation. The data were recorded over a 2*θ* range of 10 to 90°.

### Catalytic Reaction

Catalytic activity tests for the DRM were performed using a flow-type reactor, MicrotracBEL BELCAT II. Prior to reaction, the catalyst (100 mg) was loaded in a tubular reactor (inner diameter = 7.5 mm) and fixed with quartz wool on both sides. Then, the catalyst was reduced by H_2_ at 30 mL/min flow at 450 °C for 2 h. The DRM was conducted at 550 °C under a mixed gas stream of CO_2_/CH_4_/N_2_ = 10/10/5 (*v*/*v*/*v*) with a total gas flow rate of 25 mL/min. The temperature of the reactor was maintained at 550 °C for 15 h. The gaseous products were analyzed using a gas chromatograph (GL Sciences GC3200) with an active carbon column equipped with a TCD.

The CH_4_ and CO_2_ turnover frequencies (TOFs) were calculated by the moles of CH_4_ or CO_2_ converted per second per the moles of catalysts with the following equation:

$${\text{TOF }} = \frac{{N ({\text{gas}})}}{{N ({\text{catalyst}})}} \times {\text{conversion}}$$

where *N* (gas), *N* (catalyst), and conversion represent gas flow rate (mol/s), amount of catalyst (mol), and conversion of reactant gas, respectively.

## Results and Discussion

### Catalyst Preparation

**Fig. 2:**
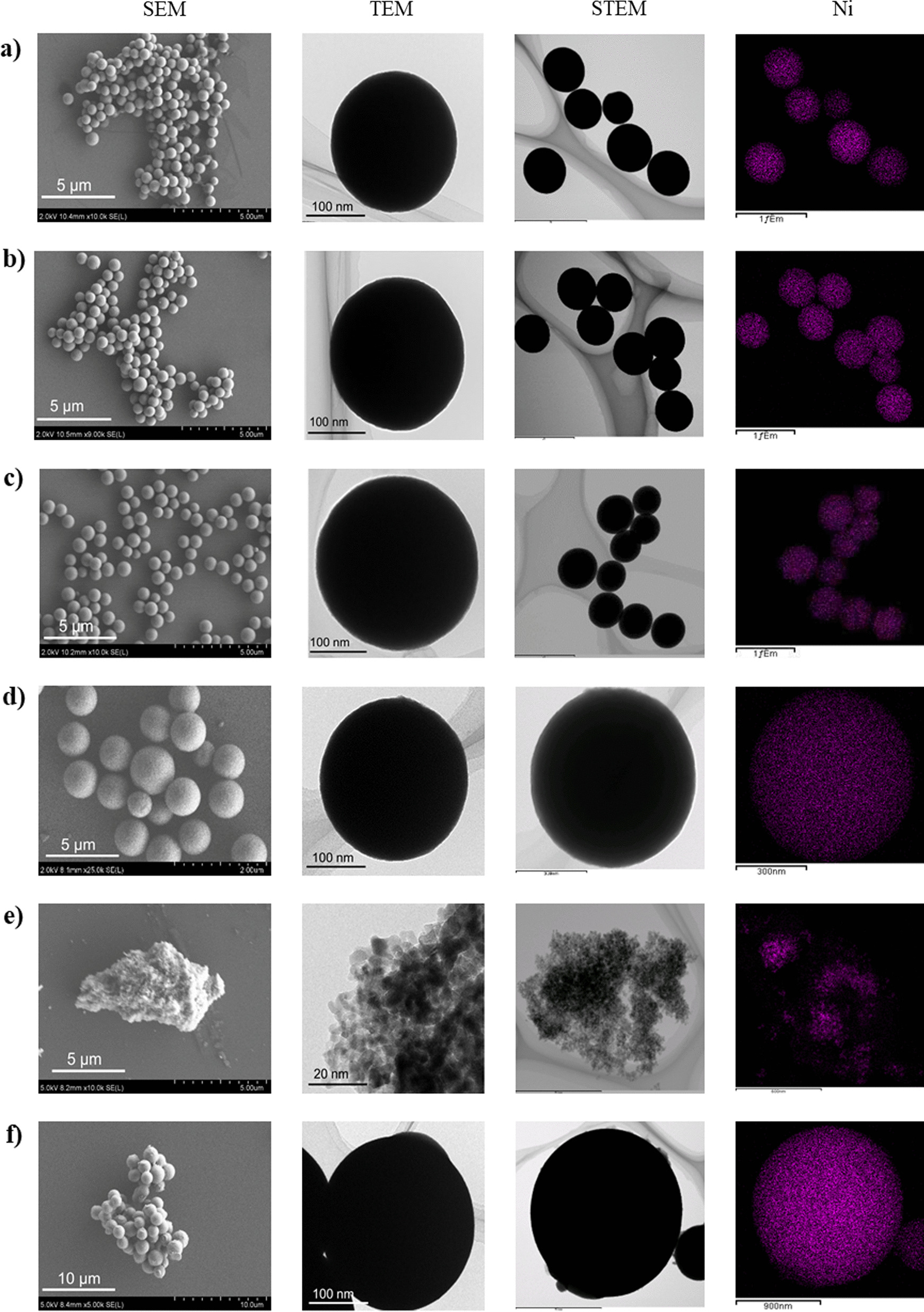
SEM, TEM, and STEM/EDX images of as-prepared Ni catalysts embedded in large ZrO_2_-based supports and TEM images of them after hydrogen reduction: **a** *L*-Ni@ZrO_2_, **b** *L*-Ni@SiO_2_-ZrO_2_, **c** *L*-Ni@MgO-ZrO_2_, and **d** *L*-Ni@Y_2_O_3_-ZrO_2_. SEM and TEM images of Ni catalysts prepared by impregnation method: **e** *U*-Ni/ZrO_2_ and **f** *L*-Ni/ZrO_2_

The *impregnation method* is a simple and representative preparation technique that affords supported nanometal catalysts. In addition, it is a versatile technique, applicable to almost all types of supports. We applied this technique to obtain a Ni catalyst supported on commercially available UEP-100 ZrO_2_ NPs, yielding *U*-Ni/ZrO_2_. A ZrO_2_ porous sphere supported Ni catalyst (*L*-Ni/ZrO_2_) was also prepared by the impregnation method [22]. The estimated secondary particle sizes, specific surface areas, pore volumes, and elemental content of the obtained materials are summarized in Table 1. The Ni contents of the catalysts (*U*-Ni/ZrO_2_: 8.2 wt%) and (*L*-Ni/ZrO_2_: 8.4 wt%) were close to the Ni contents in the 10.0 wt% precursor solutions. Judging from the SEM and TEM images (Fig. 2e), *U*-Ni/ZrO_2_ was a simple aggregate of primary NPs with a specific surface area of 76 m^2^/g after calcination in air, while *L*-Ni/ZrO_2_ exhibited a spherical morphology (Fig. 2f) with a specific surface area of 20 m^2^/g. The XRD patterns of *U*-Ni/ZrO_2_ (Fig. 4i) and *L*-Ni/ZrO_2_ (Fig. 4j) correspond to monoclinic and cubic ZrO_2_, respectively, and no peaks corresponding to cubic Ni were observed. The broad peak width of the ZrO_2_ signals of *L*-Ni/ZrO_2_ clearly indicate that the ZrO_2_ crystallite sizes in *L*-Ni/ZrO_2_ are very small (< 5 nm, according to the Scherrer equation). In the case of *L*-Ni/ZrO_2_, high-temperature H_2_ reduction at 450 °C for 2 h caused narrowing of the ZrO_2_ peaks, and a new peak corresponding to the cubic Ni phase appeared (Fig. 4Bj). These results clearly indicate that sintering of the ZrO_2_ primary particles and Ni particles occurred. Meanwhile, the *coprecipitation technique* is an alternative way to prepare noble metal catalysts supported on base metal oxides [23]. Typically, a mixed solution of a noble metal salt and a base metal salt is neutralized by an alkaline solution to yield a coprecipitate containing mixed hydroxides of both metals. The coprecipitate is calcined to produce a noble metal catalyst supported on a base metal oxide. We applied the fundamentals of the coprecipitation technique to the solvothermal reaction. A precursor solution containing Ni(NO_3_)_2_ and Zr(O^*n*^Bu)_4_ in ethanol was treated solvothermally at 250 °C to yield *L*-Ni@ZrO_2_, in which a Ni catalyst was supported on ZrO_2_ porous spheres. The product was calcined at 300 °C for 2 h in air to remove organic residues. Our proposed mechanism of the solvothermal reaction is as follows: Ni(NO_3_)_2_ and Zr(O^*n*^Bu)_4_ can be hydrolyzed by H_2_O, which is generated through the condensation of ethanol at high temperature, to yield hydroxides of both metals. At elevated temperatures, they condense with each other, yielding a mixture of corresponding metal oxides at the nanoscale. Then, nickel hydroxide and/or nickel oxide in the mixed oxides can be reduced by high-temperature ethanol under solvothermal conditions, yielding embedded Ni metal NPs in ZrO_2_ porous sphere supports.

**Table 1:** Specific surface area, pore volume, secondary particle size, and element contents of Ni catalyst supported on ZrO_2_-based porous spheres

Materials^1)^	Secondary Particle size^2)^ (nm)	Specific surface area^3)^ (m^2^/g)		Pore volume^4)^ (cm^3^/g)	Element content^5)^				
		Before H_2_ reduction	After H_2_ reduction		Ni^5,6)^	Si^5,7)^	Mg^5,7)^	Y^5,7)^	Zr^5,7)^
*L*-Ni@ZrO_2_	602 ± 63	71	4	< 0.1	9.5	–	–	–	100
*L*-Ni@SiO_2_-ZrO_2_	700 ± 61	135	2	< 0.1	8.4	5.6	–	–	94.4
*L*-Ni@MgO-ZrO_3_	618 ± 59	114	6	0.18	8.9	–	11.9	–	88.1
*L*-Ni@Y_2_O_3_-ZrO_2_	710 ± 120	238	4	0.88	6.5^8)^	–	–	9.0^8)^	91.0^8)^
*S*-Ni@ZrO_2_	120 ± 50	179	140	0.87	9.0	–	–	–	100
*S*-Ni@SiO_2_-ZrO_2_	63 ± 18^9)^	269	196	0.47	9.0	6.1	–	–	93.9
*S*-Ni@MgO-ZrO_2_	83 ± 25	167	151	0.28	6.6	–	11.5	–	98.5
*S*-Ni@Y_2_O_3_-ZrO_2_	115 ± 36	267	130	0.35	6.5^8)^	–	–	9.6^8)^	90.4^8)^
*U*-Ni/ZrO_2_^10)^	^11)^	76	73	0.36	8.2	–	–	–	–
*L*-Ni/ZrO_2_^12)^	1340 ± 130	20	3	< 0.1	8.4	–	–	–	–

1) Materials were used after calcination in air at 300 °C for 1 h. 2) As prepared materials. Evaluated by SEM images of at least 100 particles. 3) Brunauer–Emmett–Teller method. 4) BJH method. 5) Fluorescent X-ray analysis. 6) Weight %. 7) Atomic ratio of Si, Mg, or Y *vs.* Zr. 8) ICP-OES method. 9) Evaluated by TEM images of at least 100 particles. 10) Ni NPs were supported on commercially available ZrO_2_ (UEP-100) by impregnation method. 11) Not measured. 12) Ni NPs were supported by an impregnation on ZrO_2_ porous spheres obtained by a solvothermal method

SEM and TEM images of the products revealed that *L*-Ni@ZrO_2_ had an almost perfect spherical morphology with a diameter of 602 ± 63 nm (Fig. 2a). STEM/EDX (Additional file 1: Fig. S1) and XRF analyses indicate perfect dispersion of Ni atoms throughout the spheres with 9.5 wt% content. The XRD spectrum of *L*-Ni@ZrO_2_ indicates that ZrO_2_ has a cubic crystal phase with a very small crystallite size (< 5 nm, according to the Scherrer equation), while no peaks related to Ni species were observed, indicating the small particle size of Ni (Fig. 4Aa). H_2_ reduction of *L*-Ni@ZrO_2_ at 450 °C for 2 h resulted in narrower peaks for ZrO_2_, and a peak corresponding to the cubic Ni phase (Fig. 4Ba). The specific surface area changed from 71 to 4 m^2^/g, which, unfortunately, indicates that H_2_ reduction at high temperature caused considerable sintering of the ZrO_2_ NPs and Ni NPs.

According to similar solvothermal reactions, Ni catalysts embedded in ZrO_2_ composites, *L*-Ni@SiO_2_-ZrO_2_, *L*-Ni@MgO-ZrO_2_, and *L*-Ni@Y_2_O_3_-ZrO_2_, were easily obtained from the precursor solutions including additional tetraethoxy silane, magnesium acetylacetonate, and yttrium isopropoxide, respectively. SEM and TEM images show that the obtained secondary particles have spherical morphology with 600–700 nm diameters (Fig. 2b–d). The STEM/EDX results indicate that Ni, Si, Mg, and Y atoms were dispersed homogeneously throughout the network of porous spheres (Additional file 1: Fig. S1). XRF or ICP-OES analysis revealed that the embedded Ni contents of the catalysts obtained by the solvothermal method were more than 8 wt% in the cases of *L*-Ni@SiO_2_-ZrO_2_ and *L*-Ni@MgO-ZrO_2_, while *L*-Ni@Y_2_O_3_-ZrO_2_ was slightly smaller, at 6.5 wt%. The Si content was ca. 6 wt%, and the Mg and Y content was 9–12 wt%, which is similar to the corresponding content (10 wt%) in the precursor solutions. Moreover, *L*-Ni@SiO_2_-ZrO_2_, *L*-Ni@MgO-ZrO_2_, and *L*-Ni@Y_2_O_3_-ZrO_2_ exhibited higher specific surface areas (110–240 m^2^/g) than that of the prototype *L*-Ni@ZrO_2_ (71 m^2^/g). However, the high specific surface areas were drastically reduced to 2–6 m^2^/g, when the catalysts were reduced by H_2_ at 450 °C for 2 h. Unlike the results for *L*-Ni@MgO-ZrO_2_, in the *L*-Ni@SiO_2_-ZrO_2_ and *L*-Ni@Y_2_O_3_-ZrO_2_ results exhibited almost no peaks related to Ni species after high-temperature reduction, indicating that the ZrO_2_ and Ni particles remained small even after H_2_ reduction at 450 °C.

**Fig. 3:**
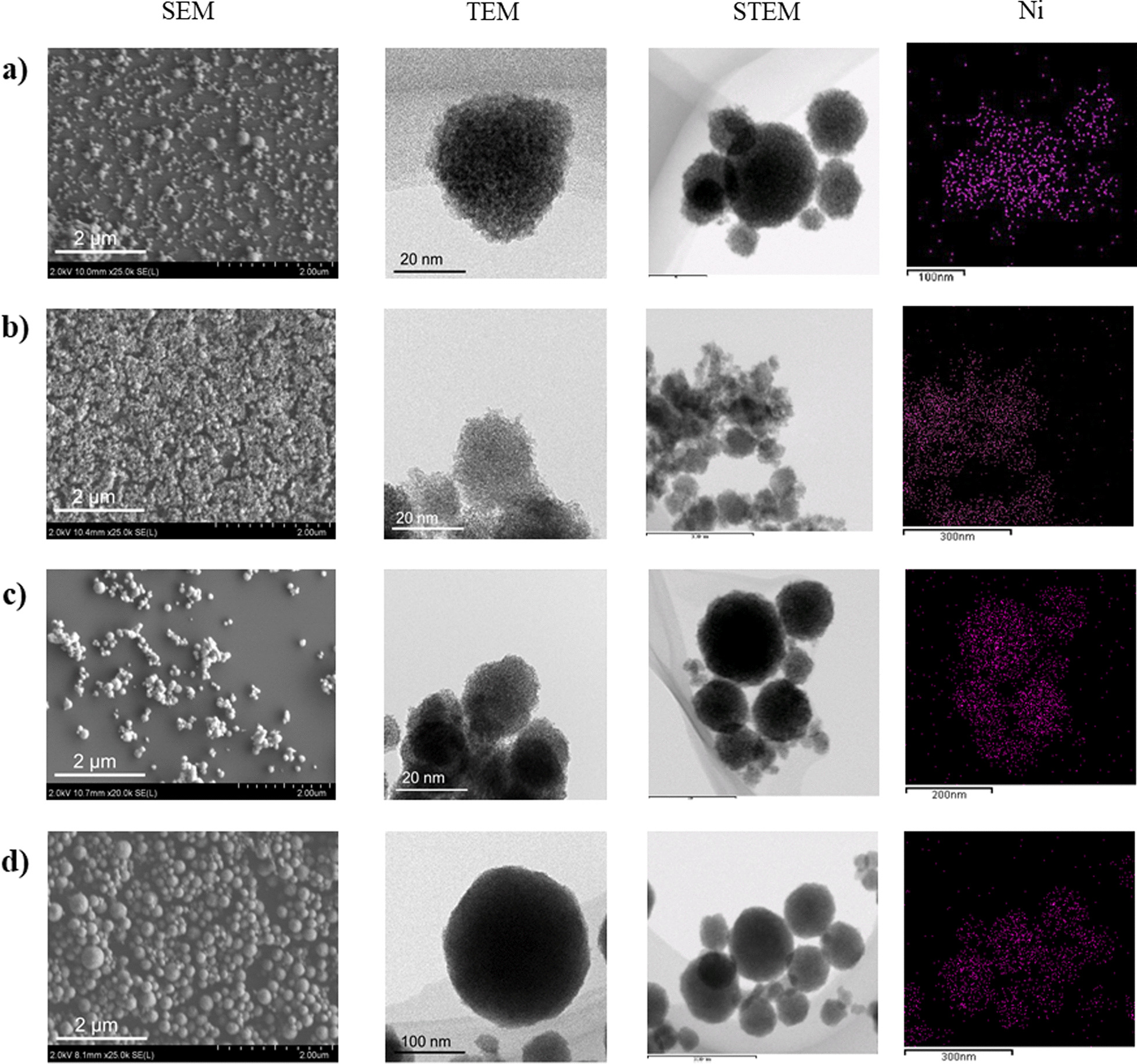
SEM, TEM, and STEM/EDX images of as-prepared Ni catalysts embedded in small ZrO_2_-based supports and TEM images of them after hydrogen reduction: **a** *S*-Ni@ZrO_2_, **b** *S*-Ni@SiO_2_-ZrO_2_, **c** *S*-Ni@MgO-ZrO_2_, and **d** *L*-Ni@Y_2_O_3_-ZrO_2_

**Fig. 4:**
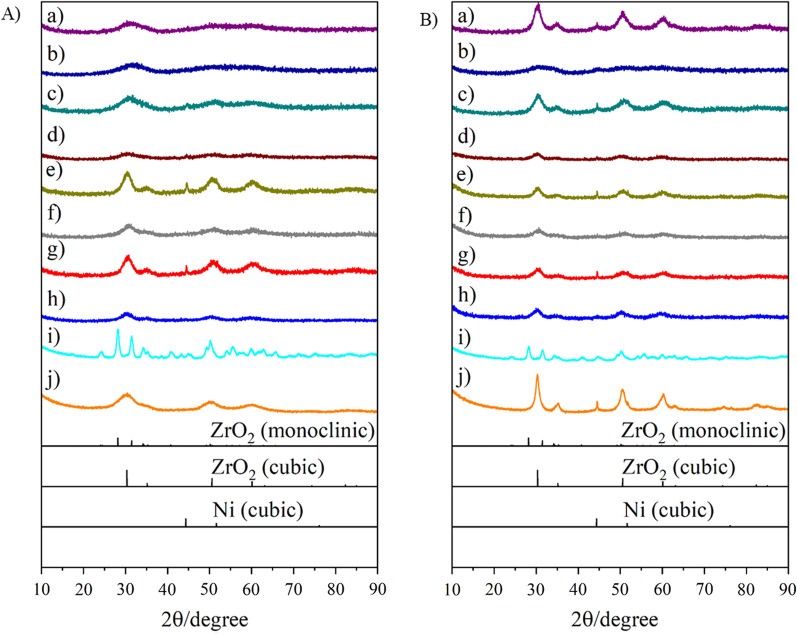
XRD patterns of Ni catalysts embedded in ZrO_2_-based supports: a) *L*-Ni@ ZrO_2_, b) *L*-Ni@SiO_2_-ZrO_2_, c) *L*-Ni@MgO-ZrO_2_, d) *L*-Ni@Y_2_O_3_-ZrO_2_, e) *S*-Ni@ZrO_2_, f) *S*-Ni@SiO_2_-ZrO_2_, g) *S*-Ni@MgO-ZrO_2_, h) *S*-Ni@Y_2_O_3_-ZrO_2_, i) *U*-Ni/ZrO_2_, and j) *L*-Ni/ZrO_2_. **A** Before H_2_ reduction and **B** after H_2_ reduction

Thus, we thought that the porous spheres consisting of slightly larger ZrO_2_ primary particles would tolerate sintering even at high temperatures, and smaller (spherical) secondary particles would be effective for gas diffusion into and out of porous sphere catalysts. Therefore, the reaction conditions, such as solvent, reaction temperature, and reaction time, were optimized to produce small (spherical) secondary particles with slightly larger primary particle. Finally, we found that the addition of a small volume of water to the precursor solutions dramatically reduced the secondary particle sizes from to 600–700 nm to 60–120 nm (Figs. 2 and 3). The Ni catalysts embedded in the *small* porous supports are denoted *S*-Ni@ZrO_2_, *S*-Ni@SiO_2_-ZrO_2_, *S*- Ni@MgO-ZrO_2_, and *S*-Ni@Y_2_O_3_-ZrO_2_. Judging from the SEM and TEM images, all the catalysts exhibited small secondary particle sizes of 63–120 nm (Fig. 3). The Ni atom is dispersed homogeneously throughout the (composite) supports in all cases (Additional file 1: Fig. S2) at 6.5–9.0 wt%.As shown in Fig. 4Aa–h, the XRD peaks for ZrO_2_ in the small porous spheres, *S*-Ni@ZrO_2_, *S*-Ni@SiO_2_-ZrO_2_, *S*-Ni@MgO-ZrO_2_, and *S*-Ni@Y_2_O_3_-ZrO_2_, are somewhat sharper than those of the large porous spheres, *L*-Ni@ZrO_2_, *L*-Ni@SiO_2_-ZrO_2_, *L*-Ni@MgO-ZrO_2_, and *L*-Ni@Y_2_O_3_-ZrO_2_, indicating that the crystallite size of the ZrO_2_ primary particles in the small porous spheres slightly increased as expected. Clear Ni peaks were also observed for *S*-Ni@ZrO_2_ and *S*-Ni@MgO-ZrO_2_ (Fig. 4Ae,g), while almost no Ni peaks were observed in the cases of *S*-Ni@SiO_2_-ZrO_2_ and *S*-Ni@Y_2_O_3_-ZrO_2_ (Fig. 4Af,h). Notably, the XRD profiles of the four samples were very broad (Fig. 4Ae–h), and a cubic Ni phase was observed in the cases of *S*-Ni@ZrO_2_ and *S*-Ni@MgO-ZrO_2_. Almost no Ni peaks were observed in the cases of *S*-Ni@SiO_2_-ZrO_2_ (Fig. 4Bf) and *S*-Ni@Y_2_O_3_-ZrO_2_ (Fig. 4Bh), even after H_2_ reduction at 450 °C for 2 h, suggesting that the small catalysts are high-temperature-resistant catalysts.

### DRM Reaction

To elucidate the effect of the embedded morphology of Ni NPs on ZrO_2_-based porous spheres, the DRM was selected as a probe reaction. Generally, the DRM is performed at temperatures over 700 °C to suppress carbon deposition on the catalyst metal NPs. However, here, we intentionally selected a lower temperature (550 °C) to investigate the suppression effects resulting from the particular morphology of our Ni NPs in their catalyst framework on carbon deposition.

**Fig. 5:**
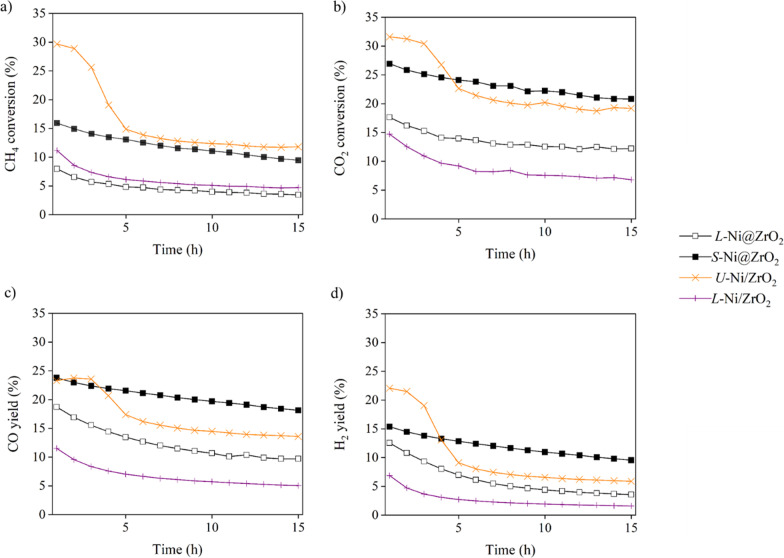
CH_4_ and CO_2_ conversions; CO and H_2_ yields of DRM reactions catalyzed by *U*-Ni/ZrO_2_, *L*-Ni/ZrO_2_, *L*-Ni@ZrO_2_, and *S*-Ni@Y_2_O_3_-ZrO_2_

Figure 5 shows the results of the DRM reaction catalyzed by *U*-Ni/ZrO_2_, *L*-Ni/ZrO_2_, *L*-Ni@ZrO_2_, and *S*-Ni@ZrO_2_. Notably, *U*-Ni/ZrO_2_ prepared by impregnation from commercially available UEP-100 ZrO_2_ demonstrated the highest catalytic ability in both CO_2_ and CH_4_ conversions, and the highest CO and H_2_ yields at the very beginning of the reaction. However, the catalytic ability was rapidly lost (within 5 h). *L*-Ni/ZrO_2_ and *L*-Ni@ZrO_2_ were prepared by impregnation from ZrO_2_ porous spheres and solvothermal method, respectively. Those catalysts exhibited relatively lower catalytic abilities in both CO_2_ and CH_4_ conversions, and CO and H_2_ yields. However, small *S*-Ni@ZrO_2_ particles exhibited high activity in CH_4_ and CO_2_ conversions as well as high CO and H_2_ yields compared to those of the impregnated *L*-Ni/ZrO_2_ and embedded *L*-Ni@ZrO_2_. These differences can be ascribed to the much higher specific surface area of *S*-Ni@ZrO_2_ (140 m^2^/g, after H_2_ reduction) than those of *L*-Ni/ZrO_2_ (3 m^2^/g, after H_2_ reduction) and *L*-Ni@ZrO_2_ (4 m^2^/g, after H_2_ reduction). Turn over frequencies (TOFs) of the catalysts are summarized in Table 2. The values were comparable to a representative low-temperature DRM catalyzed by Ni catalyst supported on SiO_2_-ZrO_2_ obtained by co-impregnation method [24].

**Table 2:** TOF for 550 ℃ DRM reaction^1)^

Catalyst	TOF (× 10^3^ s^−1^)	
	CH_4_	CO_2_
*S*-Ni@ ZrO_2_	5.7	11
*S*-Ni@SiO_2_-ZrO_2_	9.6	14
*S*-Ni@MgO-ZrO_2_	7.0	11
*S*-Ni@Y_2_O_3_-ZrO_2_	8.4	13

1) Evaluated by conversion of reactant gas and Ni amount

### Carbon Suppression Behavior in DRM

**Fig. 6:**
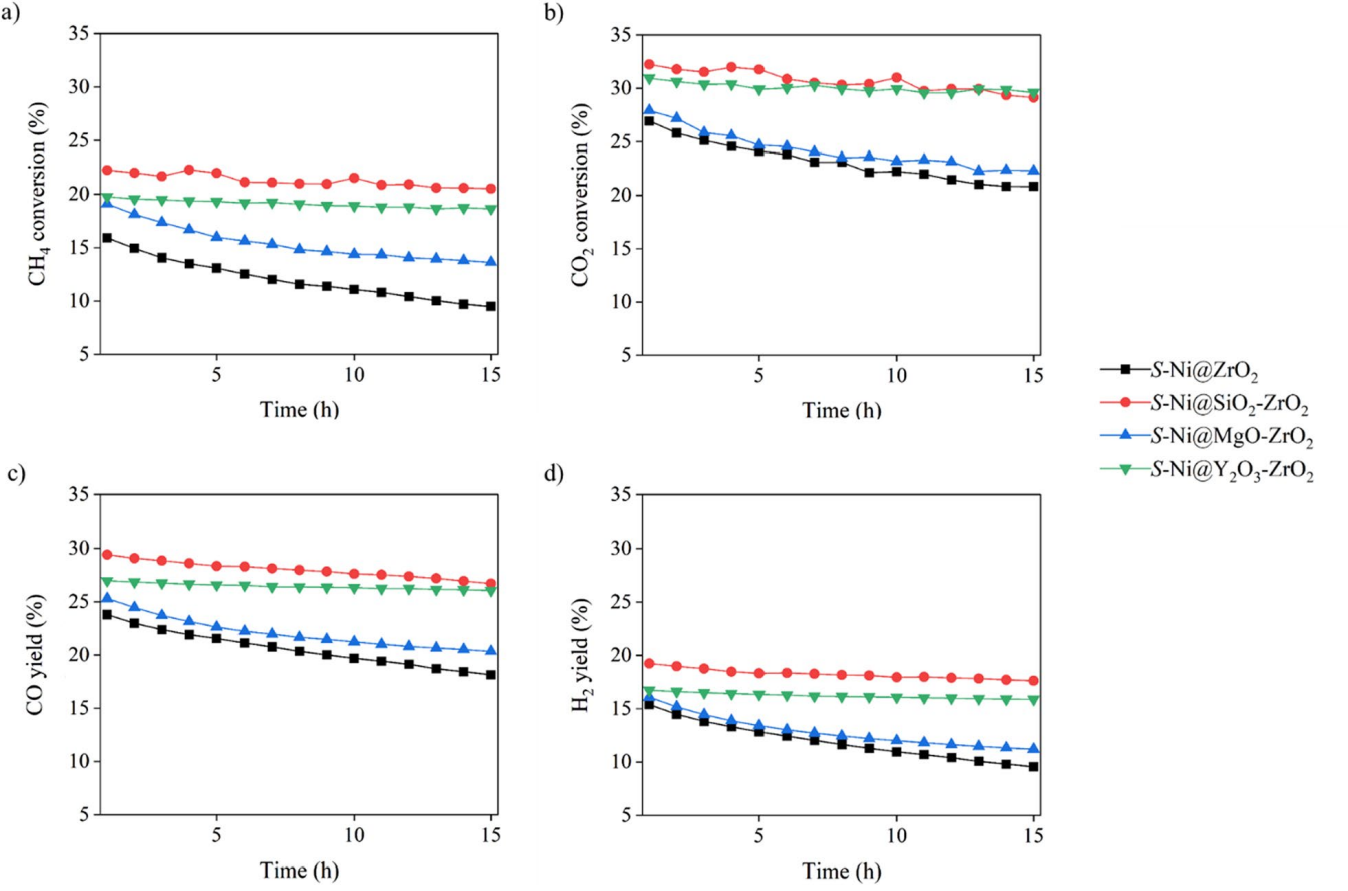
CH_4_ and CO_2_ conversions; CO and H_2_ yields of DRM reactions catalyzed by *S*-Ni@ZrO_2_, *S*-Ni@SiO_2_-ZrO_2_, *S*-Ni@MgO- ZrO_2_, and *S*-Ni@Y_2_O_3_-ZrO_2_

We abandoned the large catalysts and selected the small catalysts for the DRM. As shown in Fig. 6, the Ni catalysts embedded in the ZrO_2_ composite (*S*-Ni@SiO_2_-ZrO_2_, *S*-Ni@MgO-ZrO_2,_ and *S*-Ni@Y_2_O_3_-ZrO_2_) exhibited better performance than the prototype *S*-Ni@ZrO_2_. The results can be classified into two groups: One group includes *S*-Ni@ZrO_2_ and *S*-Ni/MgO-ZrO_2_, which exhibit lower performance, and the other includes *S*-Ni@SiO_2_-ZrO_2_ and *S*-Ni@Y_2_O_3_-ZrO_2_, which exhibit higher performance. The conversion of CH_4_ and CO_2_ and the CO and H_2_ yields in the reactions catalyzed by *S*-Ni@SiO_2_-ZrO_2_ and *S*-Ni@Y_2_O_3_-ZrO_2_ were stable for 15 h, while those catalyzed by *S*-Ni@ZrO_2_ and *S*-Ni/MgO-ZrO_2_ slowly decreased over time.

**Fig. 7:**
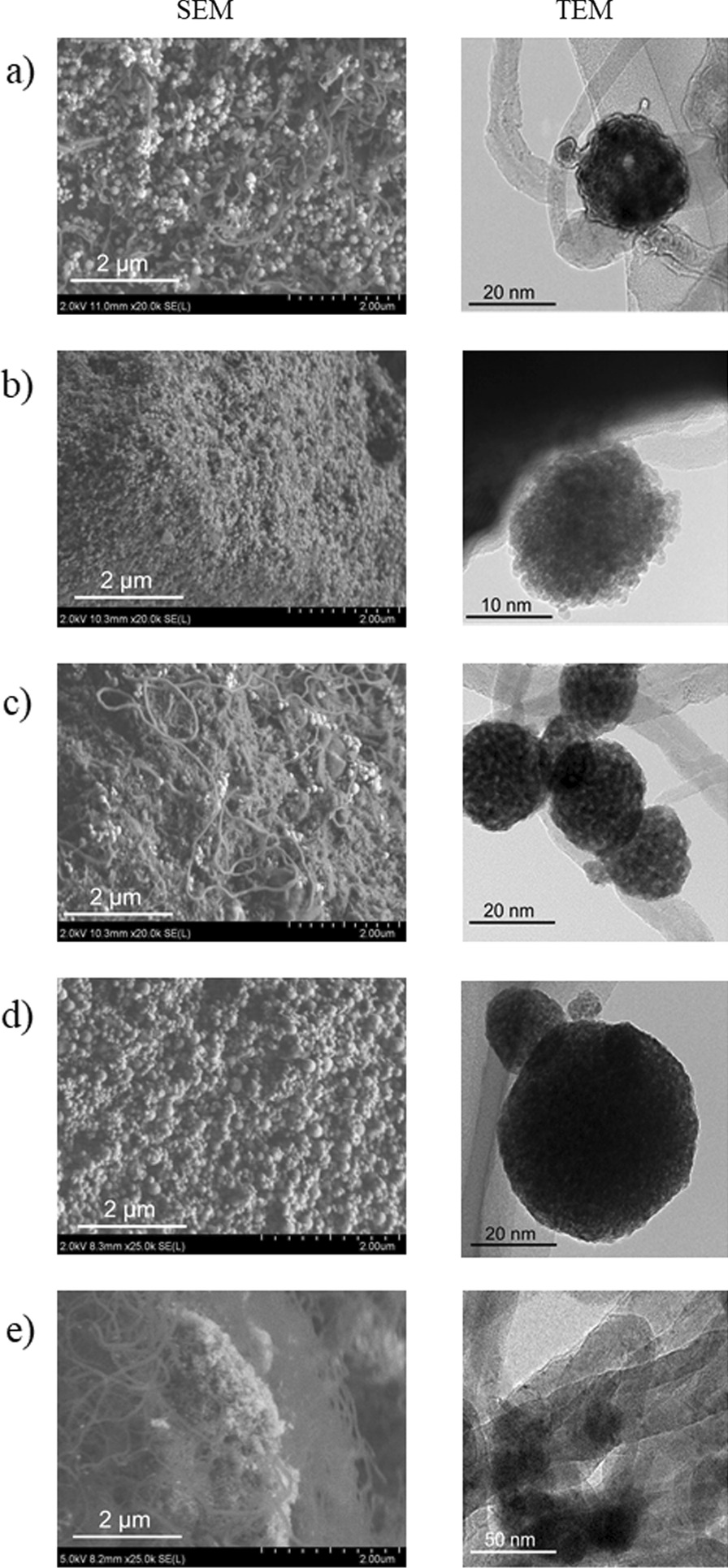
SEM and TEM images of spent Ni catalysts embedded in small ZrO_2_-based supports, **a** *S*-Ni@ZrO_2_, **b** *S*-Ni@SiO_2_-ZrO_2_, **c** *S*-Ni@MgO-ZrO_2_, **d** *S*-Ni@Y_2_O_3_-ZrO_2_, and **e** *U*-Ni/ZrO_2_

The high catalytic activity and long-term stability of *S*-Ni@SiO_2_-ZrO_2_ and *S*-Ni@Y_2_O_3_-ZrO_2_ can be ascribed to the sintering resistance of the catalysts. To directly clarify the carbon deposition on the catalysts, SEM and TEM images of the spent catalysts were obtained (Fig. 7). As expected, the formation of a large number of carbon nanotubes (CNTs) was observed in the case of prototype *S*-Ni@ZrO_2_ (Fig. 7a) and *S*-Ni@MgO-ZrO_2_ (Fig. 7c). In contrast, only a small number of CNT were observed in the cases of *S*-Ni@SiO_2_-ZrO_2_ (Fig. 7b) and *S*-Ni@Y_2_O_3_-ZrO_2_ (Fig. 7d).

**Fig. 8:**
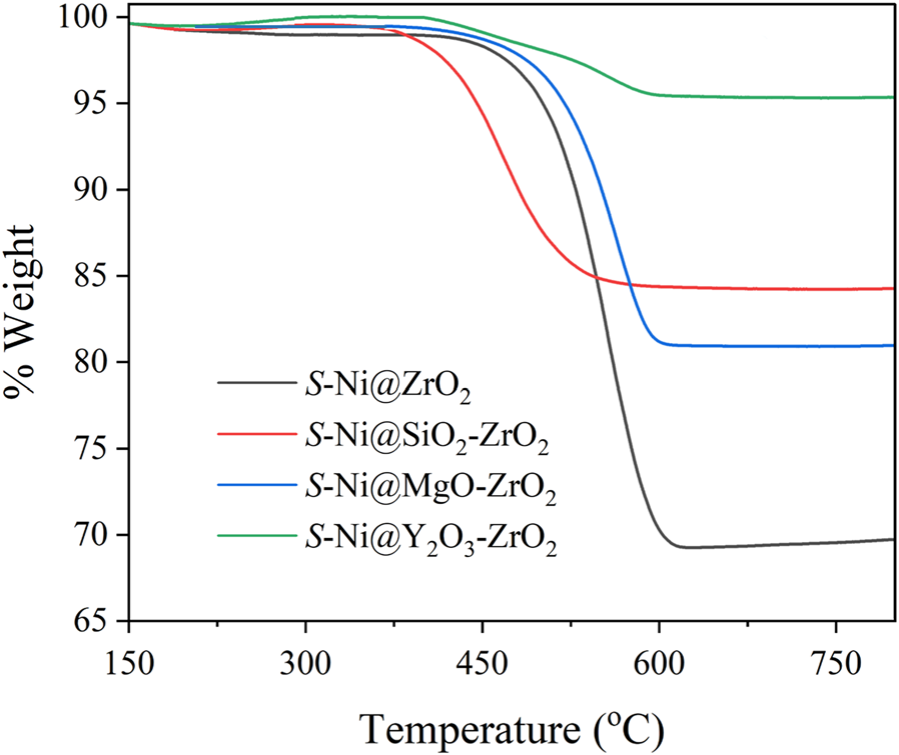
TGA profiles of spent catalysts for *S*-Ni@ZrO_2_, *S*-Ni@SiO_2_-ZrO_2_, *S*-Ni@MgO-ZrO_2_, and *S*-Ni@Y_2_O_3_-ZrO_2_

The exact quantity of deposited CNTs was estimated by TG analysis of the spent catalysts (Fig. 8). In the cases of *S*-Ni@ZrO_2_ and *S*-Ni@MgO-ZrO_2_, weight loss started at approximately 400 °C and was almost complete at 600 °C, with weight losses corresponding to 30 and 19%, respectively. However, in the cases of *S*-Ni@SiO_2_-ZrO_2_ and *S*-Ni@Y_2_O_3_-ZrO_2_, weight losses started at a lower temperature, approximately 350 °C, and had almost completed by 550 or 600 °C, with weight losses corresponding to 15 and 5%, respectively.

The different decomposition initiation temperatures suggest that the former and latter groups resulted in the formation of different carbon species. The lower decomposition temperature suggests the formation of amorphous carbon, and the higher decomposition temperature indicates the formation of CNT [25]. Thus, Ni catalysts embedded among small SiO_2_-ZrO_2_ and Y_2_O_3_-ZrO_2_ porous sphere supports effectively suppressed the formation of carbon species in the DRM reaction.

**Fig. 9:**
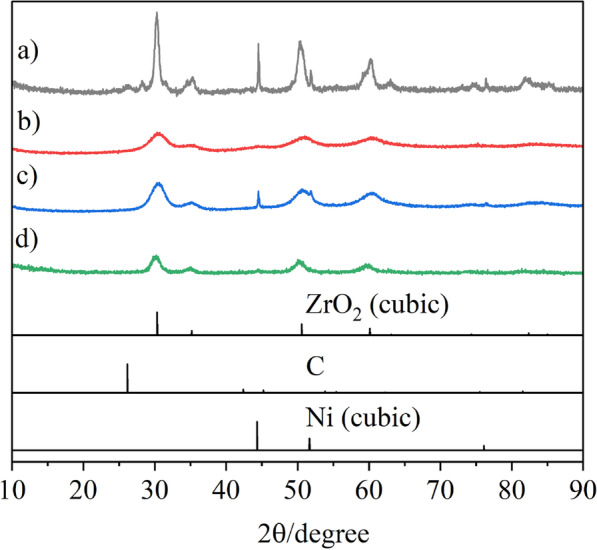
XRD patterns of spent catalysts for **a** *S*-Ni@ZrO_2_, **b** *S*-Ni@SiO_2_-ZrO_2_, **c** *S*-Ni@MgO-ZrO_2_, and **d** *S*-Ni@Y_2_O_3_-ZrO_2_

XRD spectra of the spent catalysts are shown in Fig. 9. Almost no changes were observed in the cases of *S*-Ni@SiO_2_-ZrO_2_ and *S*-Ni@Y_2_O_3_-ZrO_2_, where the profiles left broad even after DRM. However, sharper peaks corresponding to cubic ZrO_2_ and cubic Ni were recognized in the cases of *S*-Ni@ZrO_2_ and *S*-Ni@MgO-ZrO_2_. These results clearly indicate that sintering of the Ni particles and ZrO_2_ primary particles were suppressed effectively in the cases of *S*-Ni@SiO_2_-ZrO_2_ and *S*-Ni@Y_2_O_3_-ZrO_2_. Confinement effect of Ni NPs and ZrO_2_ primary particles by embedded SiO_2_ and Y_2_O_3_ can be an additional reason for the high catalytic performance of them [24].

## Conclusion

We successfully prepared embedded Ni NP catalysts in ZrO_2_ porous spheres and SiO_2_-ZrO_2_, MgO-ZrO_2_, and Y_2_O_3_-ZrO_2_ composite porous spheres by simple one-pot and single-step solvothermal reactions. The porous sphere sizes were reduced from to 600–700 nm to 63–120 nm by adding a small volume of water to the precursor solutions. The low-temperature DRM was selected as a probe reaction to verify the catalyst activity and carbon-deposition-suppression ability on the Ni NPs. The embedded Ni catalysts exhibited better catalytic activity and longer-lasting stability than the Ni-impregnated commercially available ZrO_2_ NP catalyst and Ni-impregnated ZrO_2_ porous spherical catalyst. In addition, carbon deposition on Ni NPs was suppressed by the small ZrO_2_ composites embedded with Ni NPs. In particular, the Ni catalyst embedded in small SiO_2_-ZrO_2_ porous spheres with a high specific surface area demonstrated good activity and long stability.

## Supplementary Information

**Additional file 1**. **Fig.** **S3** **a)** H_2_/CO mole ratio of DRM reactions catalyzed by *L*-Ni@ZrO_2_, *S*-Ni@ZrO_2_, *L*-Ni/ZrO_2_, and U-Ni/ZrO2. **b)** H_2_/CO mole ratio of DRM reactions catalyzed by *S*-Ni@ZrO_2_, *S*-Ni@SiO_2_-ZrO_2_, *S*-Ni@MgO-ZrO_2_, and *S*-Ni@Y_2_O_3_-ZrO_2_. **Fig. S4** SEM images of Au coated as-prepared Ni catalysts of **a)** *S*-Ni@ZrO_2_, **b)** *S*-Ni@SiO_2_-ZrO_2_, **c)** *S*-Ni@MgO-ZrO_2_, and **d)** *S*-Ni@Y_2_O_3_-ZrO_2_.

## Data Availability

The data used and analyzed during the current study are available from the corresponding authors upon reasonable request.

## Abbreviations

DRM: Dry reforming of methane
NP: Nanoparticle
MARIMO: Micro-/meso-porously architected roundly integrated metal oxides
TEM: Transmission electron microscopy
EDX: Energy-dispersive X-ray
SEM: Scanning electron microscopy
ICP-OES: Inductively coupled plasma optical emission spectroscopy
XRF: X-ray fluorescence
XRD: X-ray diffraction
TCD: Thermal conductivity detector
CNT: Carbon nanotube
