# Supplementary material for: One-Step Solvothermal Synthesis of Ni Nanoparticle Catalysts Embedded in ZrO2 Porous Spheres to Suppress Carbon Deposition in Low-Temperature Dry Reforming of Methane
Source: Nanoscale Res Lett. 2022 Apr 18;17:47. doi: 10.1186/s11671-022-03683-7 (PMC9016108; doi:10.1186/s11671-022-03683-7)
Supplement: Supplementary file 1 — Additional file 1. Fig. S3 a) H2/CO mole ratio of DRM reactions catalyzed by L-Ni@ZrO2, S-Ni@ZrO2, L-Ni/ZrO2, and U-Ni/ZrO2. b) H2/CO mole ratio of DRM reactions catalyzed by S-Ni@ZrO2, S-Ni@SiO2-ZrO2, S-Ni@MgO-ZrO2, and S-Ni@Y2O3-ZrO2. Fig. S4 SEM images of Au coated as-prepared Ni catalysts of a) S-Ni@ZrO2, b) S-Ni@SiO2-ZrO2, c) S-Ni@MgO-ZrO2, and d) S-Ni@Y2O3-ZrO2. [file 11671_2022_3683_MOESM1_ESM.docx]

Supporting Information

One-step Solvothermal Synthesis of Ni Nanoparticle Catalysts Embedded in ZrO_2_ Porous Spheres to Suppress Carbon Deposition in Low-temperature Dry Reforming of Methane

Meiliefiana Meiliefiana^1^, Tsuzumi Nakayashiki^1^, Emi Yamamoto^1^, Kahoko Hayashi^1^, Masataka Ohtani^1,2*^ and Kazuya Kobiro^1,2*^

^1^ School of Environmental Science and Engineering, Kochi University of Technology, 185 Miyanokuchi, Tosayamada, Kochi, 782-8502, Japan

^2^ Research Center for Structural Nanochemistry, 185 Miyanokuchi, Tosayamada, Kochi University of Technology, Kochi, 782-8502, Japan

^*^Correspondence: [ohtani.masataka@kochi-tech.ac.jp](mailto:ohtani.masataka@kochi-tech.ac.jp);

[kobiro.kazuya@kochi-tech.ac.jp](mailto:kobiro.kazuya@kochi-tech.ac.jp)

URL: http://kobiken.jindo.com/

**
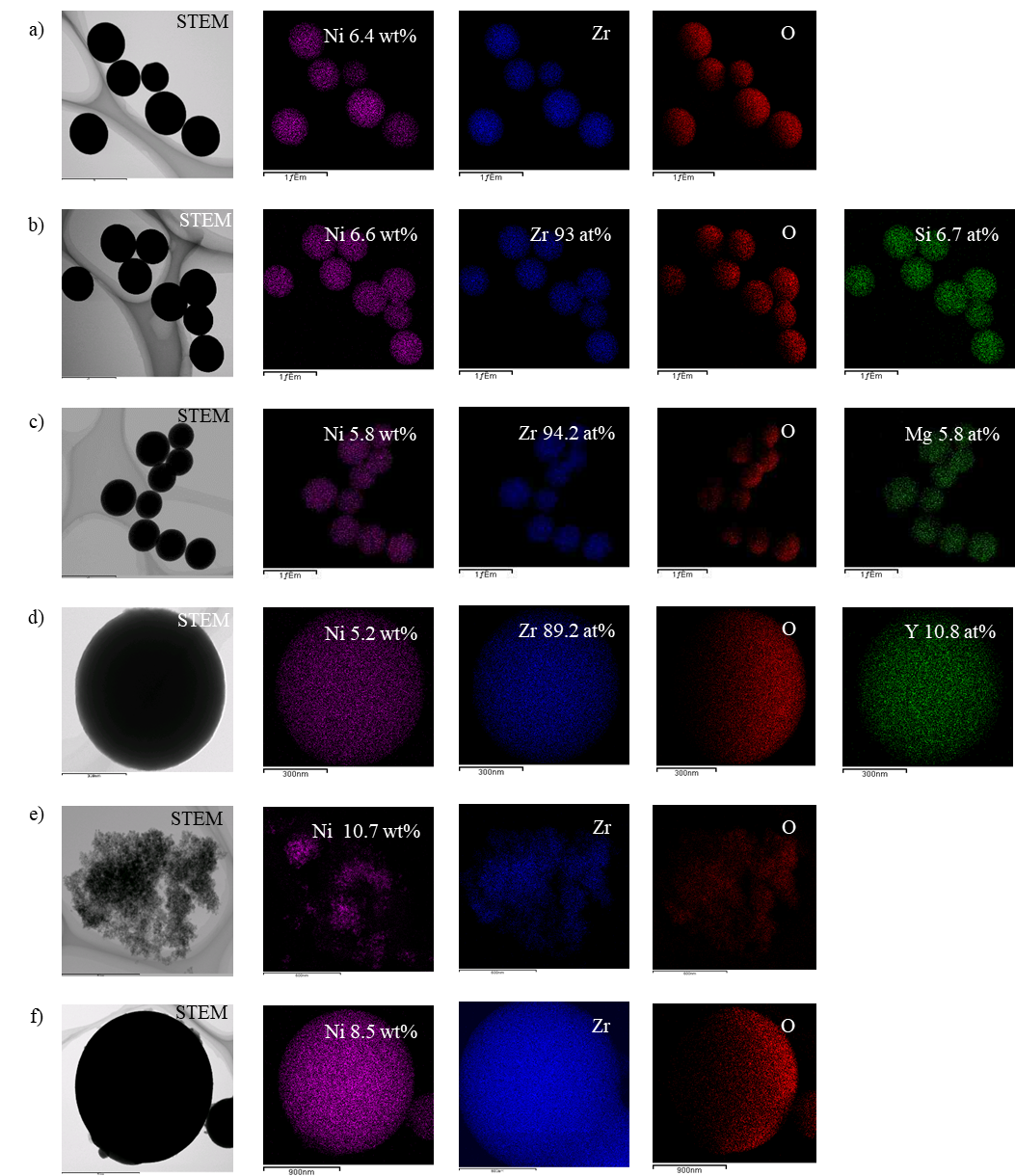
**

**Fig. S1** STEM/EDX analysis of **a)** *L*-Ni@ZrO_2,_ **b)** *L*-Ni@SiO_2_-ZrO_2_, **c)** *L*-Ni@MgO-ZrO_2_, **d)** *L*-Ni@Y_2_O_3_-ZrO_2_, **e)** *U*-Ni/ZrO_2_., and **f)** *L*-Ni/ZrO_2_

**
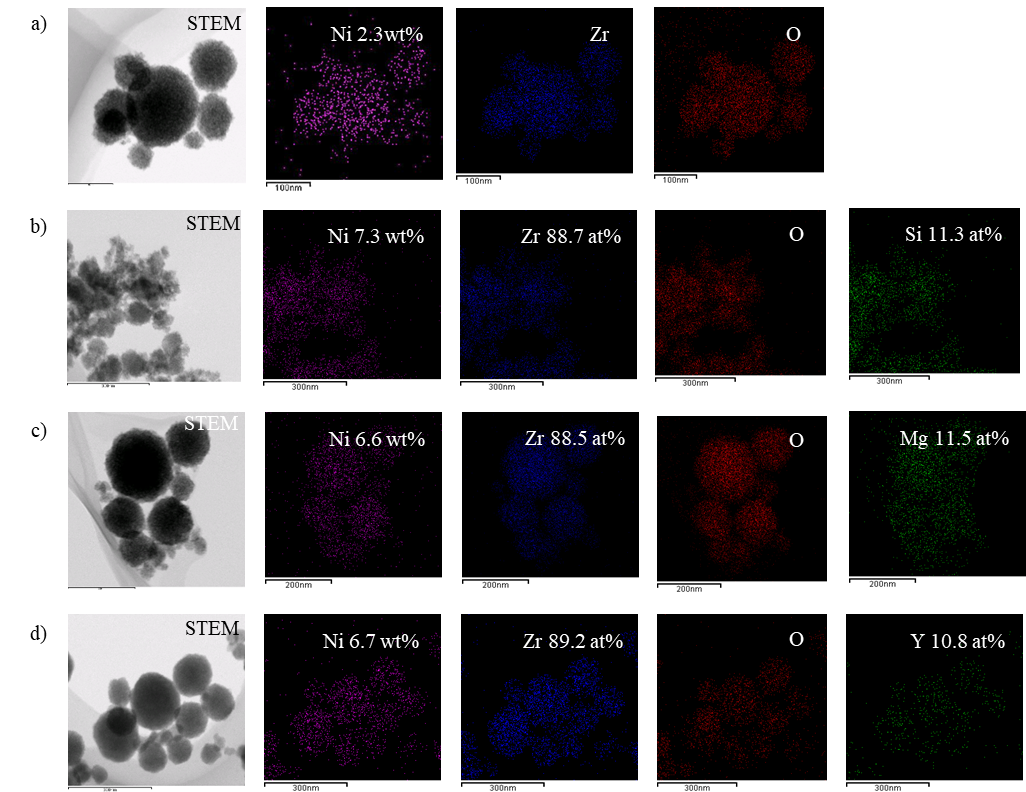
**

**Fig. S2** STEM/EDX analysis of **a)** *S*-Ni@ZrO_2,_ **b)** *S*-Ni@SiO_2_-ZrO_2_, **c)** *S*-Ni@MgO-ZrO_2_, **d)** *S-*Ni@Y_2_O_3_-ZrO_2_

**
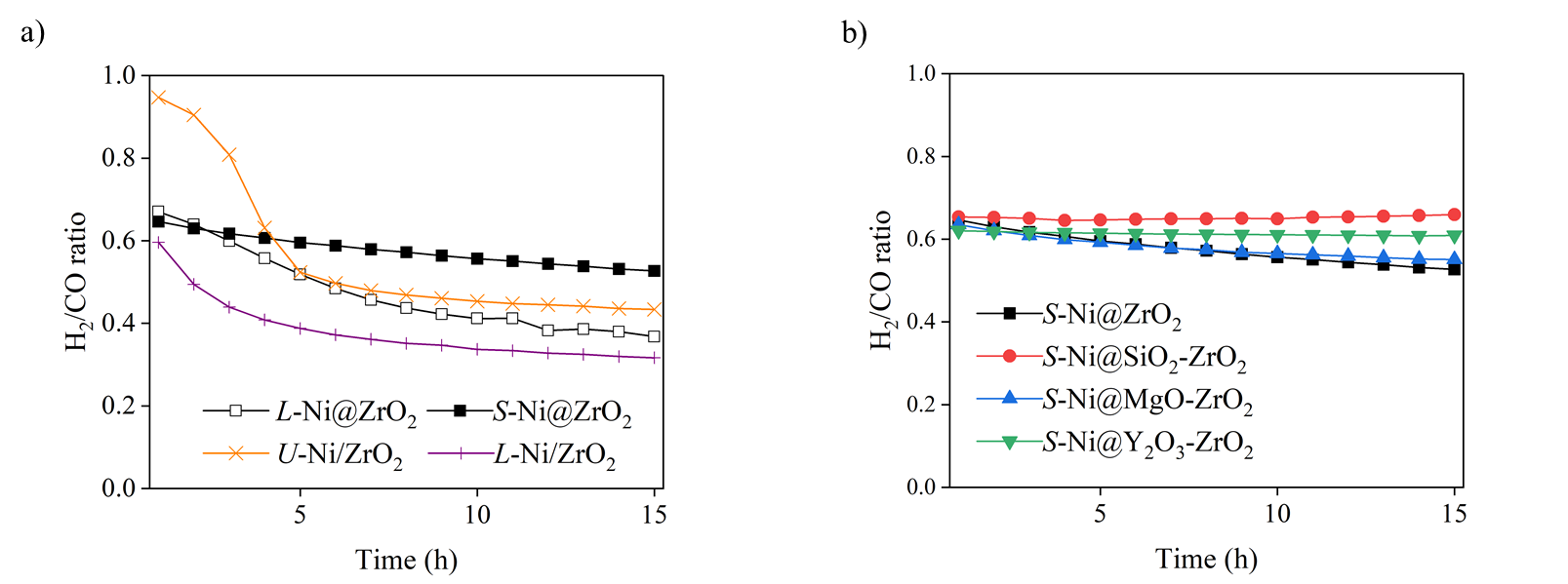
**

**Fig. S3** **a)** H_2_/CO mole ratio of DRM reactions catalyzed by *L*-Ni@ZrO_2_, *S*-Ni@ZrO_2_, *L*-Ni/ZrO_2_, and *U*-Ni/ZrO_2_. **b)** H_2_/CO mole ratio of DRM reactions catalyzed by *S*-Ni@ZrO_2_, *S*-Ni@SiO_2_-ZrO_2_, *S*-Ni@MgO-ZrO_2_, and *S*-Ni@Y_2_O_3_-ZrO_2_


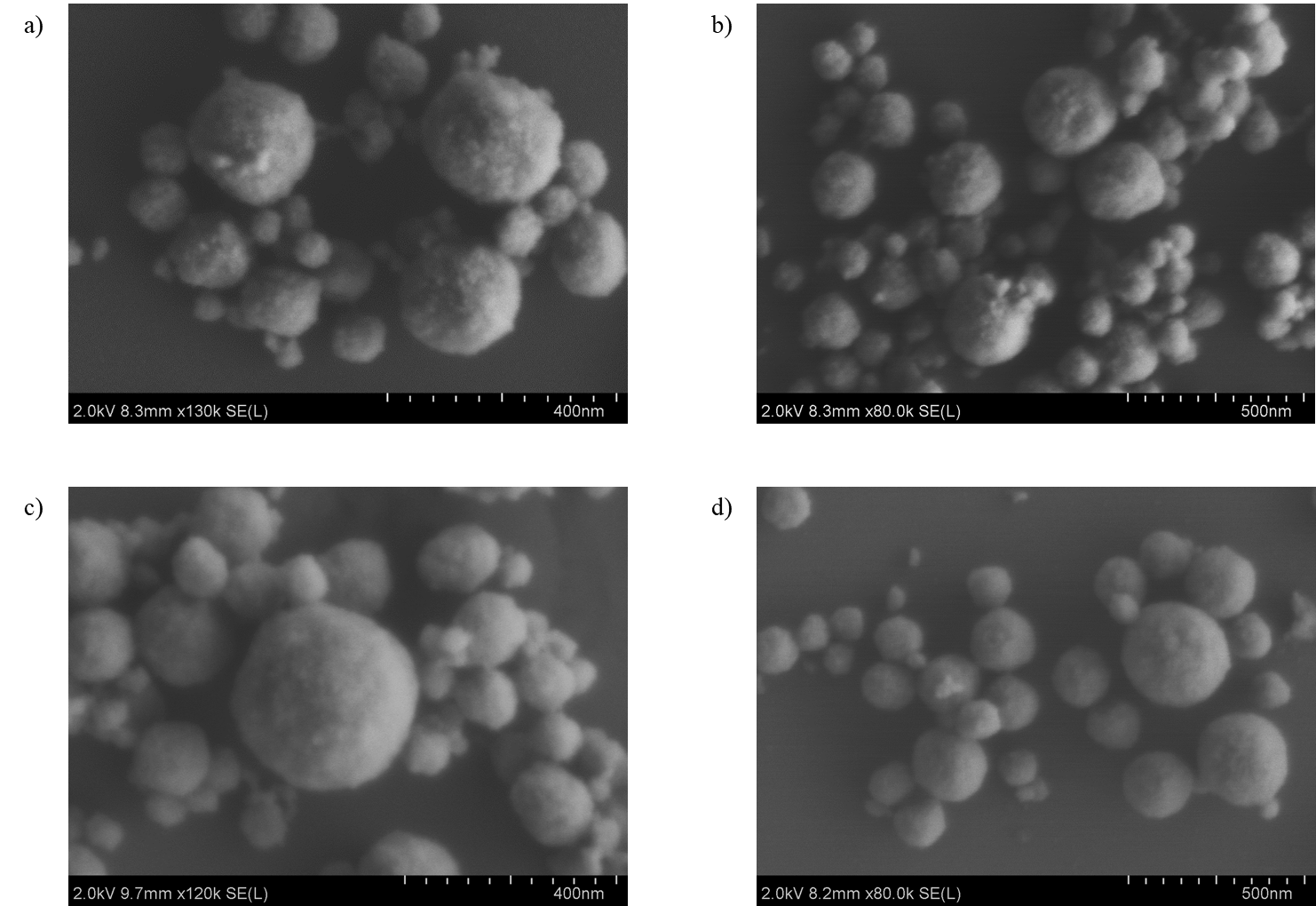


**Fig. S4** SEM images of Au coated as-prepared Ni catalysts of **a)** *S*-Ni@ZrO_2,_ **b)** *S*-Ni@SiO_2_-ZrO_2_, **c)** *S*-Ni@MgO-ZrO_2_, and **d)** *S-*Ni@Y_2_O_3_-ZrO_2_.
